# Assessment of awareness of keratoconus and its relation to eye rubbing among Saudi Arabia population

**DOI:** 10.3389/fopht.2025.1545030

**Published:** 2025-02-11

**Authors:** Nuwayyir Abdullah Alqasimi, Lujain Hatim Aljohani, Renato Ambrósio, Bader Saad AlQahtani, Nasser Saleh Al Haydar, Bdour Raja Alanazi, Danah Tariq Alfurayhan, Lina Sami Hussain Saber, Fatemah Saleh Alsalem, Nawaf Abdullah Alqahtani, Jose Manuel Vargas

**Affiliations:** ^1^ College of Medicine, Princess Nourah bint Abdulrahman University, Riyadh, Saudi Arabia; ^2^ Department of Ophthalmology, Federal University of the State of Rio de Janeiro (UNIRIO), Rio de Janeiro, Brazil; ^3^ Department of Ophthalmology, Federal University of São Paulo, São Paulo, Brazil; ^4^ Ambrosio Vision Academy, Rio de Janeiro, Brazil; ^5^ Rio Vision Hospital, Rio de Janeiro, Brazil; ^6^ Brazilian Artificial Intelligence Networking in Medicine - BrAIN, Rio de Janeiro & Maceio, Brazil; ^7^ Cornea, Cataract and Refractive Surgery Consultant, Riyadh, Saudi Arabia; ^8^ Assistant Professor at College of Medicine, Alfaisal University in Riyadh, Riyadh, Saudi Arabia; ^9^ Bachelor of Medicine and Bachelor of Surgery (MBBS), Najran, Saudi Arabia; ^10^ College of Medicine, Jouf University, Sakakah, Saudi Arabia; ^11^ College of Medicine, University of Hail, Hail, Saudi Arabia; ^12^ Faculty of Medicine, King Abdulaziz University, Jeddah, Saudi Arabia; ^13^ Bachelor of Medicine and Bachelor of Surgery (MBBS), Al-Ahsa, Saudi Arabia; ^14^ College of Medicine, King Saud bin Abdulaziz University for Health Sciences, Riyadh, Saudi Arabia; ^15^ Ophthalmology Head Section, King Abdullah Bin Abdulaziz University Hospital, Riyadh, Saudi Arabia

**Keywords:** keratoconus, eye rubbing, allergy, cornea, awareness, knowledge

## Abstract

**Background:**

Keratoconus (KC) is a bilateral, asymmetric, progressive thinning of the cornea that causes a decrease in optical quality due to induced myopia, irregular astigmatism, and higher order aberrations. KC affects 1.38 per 1,000 individuals globally, with a higher prevalence in Asian and Middle Eastern populations. Eye rubbing has been recognized as one of the leading risk factors for KC.

**Objectives:**

This study aimed to assess the knowledge and awareness about KC and its relation to eye rubbing among the population of the Kingdom of Saudi Arabia.

**Methods:**

A cross-sectional study was conducted in 2024 among people residing in different regions of Saudi Arabia. Data collection was carried out using an online questionnaire consisting of 21 questions, which were divided into two sections. Prior to administering the questionnaire, informed consent was obtained, and the confidentiality of the collected data was ensured.

**Results:**

This survey had a total of 2,059 respondents. The majority of the participants in the study were female, and their ages ranged from 18 to 30 years. Most of the participants held a university degree or higher. In total, 44% of respondents reported having allergic disorders, while 57.6% demonstrated a lack of knowledge regarding KC. The level of KC awareness was poor among Saudi residents, with 74.8% showing insufficient awareness. The group with acceptable awareness of KC was predominantly aged between 18 and 30 years and female, with a percentage of 30.4%.

**Conclusion:**

The study revealed that Saudi citizens are not knowledgeable about KC and the risks associated with eye rubbing. Additionally, the study identified several KC risk factors, including but not limited to eye rubbing, allergies, and family history. To alleviate the burden of KC in Saudi Arabia, it is imperative to enhance public health awareness and discourage the habit of eye rubbing.

## Introduction

Keratoconus (KC) is known to cause bilateral, asymmetrical corneal changes leading to progressive visual loss (1). The disease is characterized by increased sensitivity to light, eye irritation, and blurred vision. Additionally, KC can result in complications such as corneal scarring and irregular astigmatism.

Globally, KC affects 1.38 per 1,000 individuals, with Asian and Middle Eastern populations displaying an earlier onset and more severe symptoms (2). KC is a disease that has a significant burden in public health in the Middle East region, where its prevalence is 1.7% in Jordan’s population, while in Saudi Arabia, it is found to be at 2.75% in the Eastern Province and up to 4.79% among children in Riyadh (3–5).

Furthermore, KC is more prevalent in warmer nations compared to Western countries, leading to the argument that sunlight is a significant risk factor for KC, particularly in genetically susceptible individuals (1).

Although the etiology of KC remains unclear, research associates the disorder with various genetic, environmental, and mechanical factors. Previous studies suggest a link between eye rubbing, atopy, and UV exposure as triggers for KC, particularly in genetically predisposed individuals (6). KC was found to be associated with hormonal alterations, particularly a reduction of gonadotropin-releasing hormone levels; lower levels of GnRH may modulate the corneal response and thus the progression of KC (7).

The condition is also more prevalent in certain ethnic groups, such as Asians, compared to other ethnicities. Moreover, individuals with a family history of KC have been reported to show early structural indications of KC without significant visual impairment (6).

Eye rubbing has been recognized as one of the leading risk factors for KC Rubbing eyes has been identified as a major risk factor for the development of KC. Typically, this habit occurs due to eye irritation, fatigue, or psychological stress. Several studies have shown that rubbing of the eyes is a remarkable exogenous environmental trigger leading to mechanical changes in the cornea composition (1, 2). Consequently, abnormal eye rubbing has been cited as a critical factor in KC development due to its ability to cause inherent corneal mechanical damage. In Saudi Arabia, a retrospective study conducted at King Khalid Hospital in Hail city revealed that 44.8% of KC patients engaged in eye rubbing (8). The study noted that eye rubbing was the most common risk factor and accounted for nearly all cases of KC. Additionally, the mechanical impact of eye rubbing on the cornea makes individuals with KC susceptible to intraocular pressure variations and astigmatism (1).

Global awareness efforts have been initiated to educate communities about KC, its risk factors, and the importance of early screening. The Violet June Keratoconus Awareness Campaign is one of the leading initiatives spreading knowledge about KC, which implies the importance of raising awareness of this disease (9). However, KC awareness in Saudi Arabia remains suboptimal. A survey of non-medical students in Abha city revealed a widespread lack of awareness about KC (10). According to the study, over 95% of females aged 17–21 were unaware of KC. Another study conducted in Jeddah found that less than 50% of Saudi Arabian residents were aware of KC (11). The research reported that although over 70% of participants rubbed their eyes, only 30% were aware that eye rubbing could cause KC.

The literature reveals a notable gap in understanding KC and its association with eye rubbing. Additionally, there are regions in the Kingdom that remain underexplored, hindering a comprehensive understanding of KC awareness in the Saudi Arabian population. Thus, it’s important to assess the level of awareness of this disease, especially for the people who might not know that they are at high risk of having the disease or already experiencing some of its symptoms, because that will affect the treatment choices and their efficacy on the patient’s outcomes. A study evaluated the outcomes of immediate and delayed sequential bilateral corneal cross-linking (CXL) for bilateral progressive KC in two groups of patients. It was found that the group who underwent the delayed CXL exhibited progression of KC in their second untreated eye during the waiting period. This indicates the possibility of disease deterioration in untreated eyes when treatment is delayed (12).

This research aims to bridge this gap by assessing the level of awareness in Saudi Arabia and highlighting the critical role of eye rubbing as a significant risk factor.

## Methodology

### Study design and setting

A cross-sectional quantitative survey using a convenience sampling technique was conducted from March 2024 to July 2024 in Saudi Arabia to assess the level of awareness regarding KC and its relation to eye rubbing among residents from various regions of Saudi Arabia. Ethical approval was obtained from the institutional review board (IRB) of King Abdullah bin Abdulaziz University Hospital (KAAUH) (approval number: 24-0040).

Previous literature was reviewed to determine the sample size. The estimated sample size was 377 participants, calculated using the Raosoft calculator with a 5% margin of error and a 95% confidence interval. However, the final sample size was increased to 2,059 participants to address potential issues with missing data or incomplete forms.

Participants were randomly selected, and questionnaires were distributed via self-administered Google Forms. Residents of Saudi Arabia were invited to complete the questionnaire using personal contacts and social media platforms, including Twitter (Twitter Inc., San Francisco, California, United States), WhatsApp (WhatsApp LLC, Menlo Park, CA, USA), and Instagram (Meta Platforms, Inc., Menlo Park, CA, USA). Participants were informed of the study’s rationale and objectives, and informed consent was obtained. Participation was voluntary, with privacy and confidentiality fully maintained. The study included residents of Saudi Arabia aged 18 years and older.

### Survey instruments

The standardized questionnaire was constructed based on a literature review. The questionnaire was translated into Arabic and back into English to ensure accuracy. Validation of the questionnaire was tested by a review conducted by the institution’s IRB and got approved by the IRB chairman. Subsequently, a pilot survey comprising 15 subjects was carried out to test the questionnaire’s clarity and identify any omissions. Necessary changes and revisions were implemented.

#### The questionnaire was divided into two parts

1. Demographics: Questions regarding participants’ age, gender, educational level, marital status, monthly income, region of residence, profession, allergies, and other eye-related diseases. Participants were also asked about any KC diagnosis or family history of KC, as well as their eye-rubbing habits and the reasons behind them.
2. Awareness and perception: Thirteen questions assessing participants’ awareness and perception of KC, including its etiology, treatment, and reasons for eye rubbing. Participants selected the most appropriate option from the choices provided.

### Data analysis

After data collection, the datasets were reviewed, coded, and imported into the Statistical Package for the Social Sciences (SPSS), IBM SPSS Statistics for Windows, Version 22.0 (2013, IBM Corp., Armonk, New York, USA). Statistical analyses were conducted with two-tailed tests, and a p-value < 0.05 was considered statistically significant.

Each knowledge question was scored as 1 for a correct answer. Awareness was categorized as either poor or good based on a threshold score of 60%. Participants scoring less than 60% were classified as having poor awareness, while those scoring 60% or higher were considered to have good awareness.

Descriptive analyses, including frequency and percentage distribution, were performed for variables such as participants’ family history of KC, medical history of eye diseases, sources of knowledge about KC, and biographical characteristics. Awareness levels regarding KC, its risk factors, treatment methods, and general knowledge (excluding eye-rubbing practices) were analyzed using frequency tables and graphs.

To examine variations in awareness levels across individual characteristics and practices, cross-tabulation was employed. Relationships were assessed using Pearson’s chi-square test and exact probability tests for small frequency distributions.

## Results

A total of 2,059 participants took part in the study. Of these, 56.2% (*n* = 1,158) were aged 18–30 years, and 67.5% (*n* = 1,390) were female. Regarding marital status, 48.3% (*n* = 995) were married. The findings indicate that 74.1% (*n* = 1,525) had a university or higher education level, 22.5% (*n* = 464) had secondary education, and only 3.4% (*n* = 70) had an education level below secondary.

Regarding job status, 38.1% (*n* = 784) were students, 36.2% (*n* = 745) were employed, 21.7% (*n* = 447) were unemployed, and only 4% (*n* = 83) were retired. Moreover, more than half (54.2%, *n* = 1,121) earned a monthly income of less than 5,000 SAR, while 26% (*n* = 536) earned more than 10,000 SAR. Participants were distributed across different regions of Saudi Arabia as follows: 26.3% (*n* = 542) from the central region, 24.3% (*n* = 501) from the eastern region, 19.2% (*n* = 398) from the southern region, 19.1% (*n* = 393) from the western region, and 10.9% (*n* = 225) from the northern region.

In terms of family and medical history, 44% (*n* = 906) of participants reported having allergies, including nasal (14.5%), skin (11.9%), eye (7.9%), chest (6.6%), and gastrointestinal (GIT) allergies (3.1%). Additionally, 17.1% (*n* = 352) of participants had ocular diseases. Only 5.3% (*n* = 109) had been personally diagnosed with KC, while 10.5% (*n* = 217) reported having a family member diagnosed with the condition. More details are provided in **Table 1**.

**Table 1:** Demographic characteristics of the study participants and their medical history of ocular diseases (*N* = 2,059).

Parameter		Number	Percentage
Age (years)	18–30	1158	56.2
	31–39	337	16.4
	40–49	379	18.4
	≥ 50	185	9
Gender	Female	1390	67.5
	Male	669	32.5
Marital status	Married	995	48.3
	Unmarried	1064	51.7
Educational level	Below secondary	70	3.4
	Secondary	464	22.5
	University and higher	1525	74.1
Job-status	Student	784	38.1
	Unemployed	447	21.7
	Employed	745	36.2
	Retired	83	4
Monthly income (SAR)	< 5000	1121	54.2
	5000–10,000	402	19.5
	>10,000	536	26
Residential region	Central region	542	26.3
	Eastern region	501	24.3
	Northern region	225	10.9
	Southern region	398	19.2
	Western region	393	19.1
Family and medical history			
Having an allergy	Chest allergy	136	6.6
	Eye allergy	162	7.9
	Nasal allergy	299	14.5
	GIT allergy	64	3.1
	Skin allergy	245	11.9
	None	1153	56
Having ocular diseases	Yes	352	17.1
	No	1707	82.9
Diagnosed with keratoconus before	Yes	109	5.3
	No	1950	94.7
Having a family member who has been diagnosed with keratoconus	Yes	217	10.5
	No	1842	89.5

**Figure 1:**
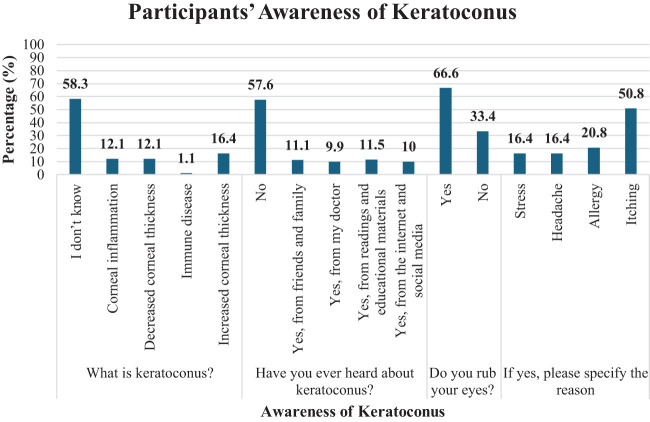
Awareness of keratoconus among the study participants.

**Figure 2:**
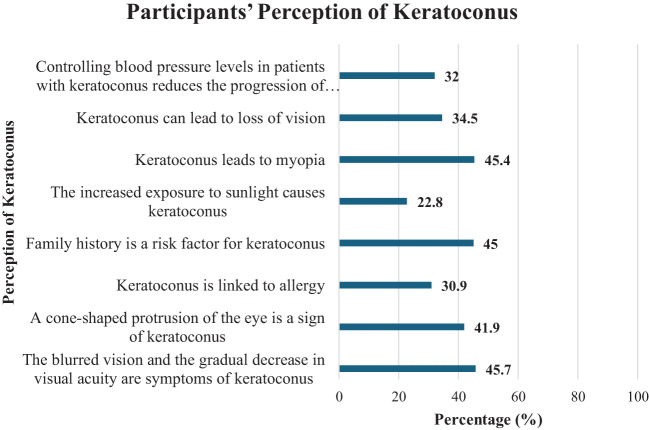
Perception of keratoconus among the study participants.

**Figures 1**, **2** illustrate the awareness and perception of the enrolled participants toward keratoconus. The results showed that the majority (58.3%, *n* = 1,201) did not know the definition of the disease, and 57.6% (*n* = 1,186) lacked awareness of keratoconus. Additionally, 66.6% (*n* = 1,371) reported rubbing their eyes, primarily due to itching (50.8%, *n* = 1,045) or allergies (20.8%, *n* = 428).

Regarding perception questions, 45.7% (*n* = 940) of participants identified blurred vision and a gradual decrease in visual acuity as symptoms of keratoconus, and 41.9% (*n* = 863) recognized a cone-shaped protrusion of the eye as a sign of the disease. Participants believed that allergy (30.9%, *n* = 636), family history (45%, *n* = 927), and sunlight exposure (22.8%, *n* = 469) were linked to keratoconus. Additionally, they thought keratoconus could lead to myopia (45.4%, *n* = 935) or vision loss (34.5%, *n* = 711).

Only a third of participants (32%, *n* = 658) knew that controlling blood pressure could help reduce the progression of the disease. Regarding treatment, most participants (30.2%, *n* = 622) considered surgery as a possible treatment for keratoconus, while only 9.5% (*n* = 196) chose eyeglasses.

The majority (76.6%, *n* = 1,577) regarded eye rubbing as an unsafe habit. Furthermore, 26.4% (*n* = 544) thought that eye allergies were the cause of eye rubbing, while 18.4% (*n* = 378) attributed it to corneal problems.

Here is the corrected and polished version of your text:

**Table 2** shows the association between the level of awareness and participants’ demographics and medical history. There is a significant association between participants’ awareness level and gender (*p* = 0.033), age, ocular disease, family history, and diagnosis of keratoconus (*p* < 0.01). Participants aged 18–30 years demonstrated a higher awareness level of keratoconus compared to those aged 40–49 years. Female participants also exhibited a higher awareness level than males.

**Table 2:** Association and distribution of awareness level based on participants’ demographics and medical history.

Factors		Awareness level N (%)		*p*-value
		Poor	Good	
Age	18–30	806 (69.6)	352 (30.4)	**< 0.01**
	31–39	272 (80.7)	65 (19.3)	
	40–49	301 (79.4)	78 (20.6)	
	≥ 50	161 (87)	24 (13)	
Gender	Female	1020 (73.4)	370 (26.6)	0.033
	Male	520 (77.7)	149 (22.3)	
Do you have any ocular diseases?	Yes	237 (67.3)	115 (32.7)	**< 0.01**
	No	1303 (76.3)	404 (23.7)	
Have you been diagnosed with keratoconus?	Yes	35 (59.6)	44 (40.4)	**< 0.01**
	No	1475 (75.6)	475 (24.4)	
Family history of keratoconus	Yes	130 (59.5)	87 (40.1)	**< 0.01**
	No	1410 (76.5)	432 (23.5)	

Bold = statistically significant results.

Regarding ocular disease and family history, participants with an ocular disease, a family history of keratoconus, or a personal diagnosis of keratoconus were more aware of the condition than those without these factors. However, there was no significant association between educational level (*p* = 0.202), allergy history (*p* = 0.346), or practicing eye rubbing (*p* = 0.634) and the level of participants’ awareness.

After applying logistic regression analysis, it was revealed that participants aged 18–30 years had significantly higher odds (3.376 times) of being aware compared to those aged 50 years and above (95% CI: 2.127–5.360, *p* < 0.01). Furthermore, participants aged 40–49 showed higher odds (1.709 times) of being aware (95% CI: 1.030–2.837, *p* = 0.038). Participants with a family history of keratoconus had significantly higher odds (2.098 times) of being aware compared to others (95% CI: 1.522–2.892, *p* < 0.01).

## Discussion

Keratoconus is an ophthalmic disorder characterized by bilateral corneal thinning. While its prevalence is often cited as 1 in 2,000, significant geographical variations exist. In Saudi Arabia, the prevalence is estimated at 4.79% among the pediatric population, while in Iran it is 2.5% (13). In the United States, Munir et al. estimate the prevalence at 0.15%, with most cases reported among males (14). Keratoconus is well known for affecting vision-related quality of life (VR-QoL) at various levels, leading to social, emotional, and physical impairments. This impact is particularly significant for keratoconus patients of working age (15).

Given its increasing prevalence and adverse impacts, this study aimed to assess keratoconus awareness and its association with eye rubbing among the population of Saudi Arabia.

Our study revealed poor keratoconus knowledge among participants. Approximately 74.8% of respondents had minimal knowledge, with only 25.2% being well informed about the disease. Awareness decreased with age; 30.4% of participants aged 18–30 years had good knowledge, while 87% of those over 50 years had poor knowledge. These findings align with other studies conducted in Saudi Arabia, where 67.5% of respondents demonstrated poor knowledge (16). Another study found that 85.7% of participants had poor knowledge, with only 14.26% being highly knowledgeable about keratoconus (2). The low awareness level extends beyond Saudi Arabia; for example, a Swiss study recorded a mean knowledge level of 35.2%, even among participants with paramedical backgrounds (17). These findings underscore the urgent need for targeted education to increase keratoconus awareness, including its prevalence, risk factors, and impacts.

Research has shown a significant association between keratoconus and eye rubbing. In this study, 66.6% of participants reported rubbing their eyes, attributing the behavior to eye allergies (26.4%), corneal problems (18.4%), ocular infections (17.8%), and keratoconus (11.9%). Other studies suggest that mechanical trauma from aggressive or prolonged eye rubbing can contribute to keratoconus development, with correlations between the intensity or frequency of eye rubbing and the disease (1, 18). A study at King Khalid Hospital identified eye rubbing as the most prevalent risk factor, affecting 44.8% of patients (1). Therefore, one effective strategy to mitigate keratoconus is the prevention of eye rubbing.

Additionally, participants in this study identified keratoconus risk factors as allergies (30.9%), family history (45%), and sunlight exposure (22.8%). Studies have suggested that atopy and allergy increase the likelihood of eye rubbing, making these conditions root causes of keratoconus (18). Several smaller studies have linked asthma, atopy, and allergy to keratoconus. Larger studies in the United States and Denmark have also highlighted allergy and asthma as significant risk factors for keratoconus (19, 20).

A prospective case-control study noted that keratoconus patients with a family history of the disease, a higher frequency of eye rubbing, or more affected family members often presented with more severe cases (21). Thus, while eye rubbing is a direct contributor, addressing root causes such as atopy is essential.

Participants in this study believed keratoconus could result in myopia (45.4%) or vision loss (34.5%). Existing research supports this, attributing the progression to central or paracentral thinning of the cornea (22). Vitar et al. also link vision loss to biomechanical weakening of the cornea (23). Beyond visual complications, keratoconus significantly impacts quality of life, causing fear of disease progression, career challenges, job loss, feelings of discrimination and isolation, and frustration with treatment outcomes (24). Even in early stages, keratoconus can substantially reduce quality of life.

The severity of these implications highlights the need for robust prevention and treatment options. In this study, 30.2% of respondents considered surgery a potential treatment for keratoconus. Studies show that intracorneal ring segment implantation is a minimally invasive method to enhance visual acuity (25). For advanced keratoconus, corneal transplantation may be necessary (26). In milder, non-progressive cases, contact lenses or spectacles may suffice, while corneal CXL is effective in stiffening the cornea during earlier stages (27). Increased awareness and knowledge would enable patients and clinicians to make timely decisions about keratoconus management (2).

Moreover, artificial intelligence (AI) holds significant potential in keratoconus awareness. AI applications could be explored and implemented to enhance public education about the disease. Improved awareness would allow patients to seek early treatment, prevent progression, and reduce economic burdens.

## Limitations

This study has several limitations. First, the participant demographics were skewed, with more females than males and over half of the participants aged between 18 and 30 years. Second, data collection was conducted via electronic questionnaires, which favored educated participants familiar with internet technologies. This is reflected in the participant demographics, as 74.1% held a university degree or higher. Also, there might be a possibility that some of them used the internet or other information resources to get knowledge about the disease.

Third, the results are susceptible to recall bias, as participants may not have accurately remembered or reported their experiences with keratoconus. Additionally, the study relied on self-reported questionnaires, which may have led some participants to provide socially desirable responses rather than truthful ones, particularly concerning their awareness levels of keratoconus.

Last, the introspective ability of participants was compromised, as they may not have accurately assessed their own behaviors, such as eye rubbing. Also, the way of eye rubbing was not specifically mentioned in our study; it should have been added to it whether the eye rubbing was by the fingertips, back of the hands, or the palm. These limitations should be considered when interpreting the findings of the study.

## Conclusion

This study aimed to assess the awareness of keratoconus and its association with eye rubbing among the population in Saudi Arabia. The findings from this cross-sectional, online survey revealed a concerning lack of awareness about keratoconus among the Saudi population. These results align with existing studies showing that many Saudis have poor knowledge of keratoconus despite its increasing prevalence.

The study identified several risk factors, including eye rubbing, allergies, and family history. Keratoconus causes various complications, such as myopia, astigmatism, and visual loss, which often necessitate surgical interventions. These findings highlight the urgent need to enhance public awareness of keratoconus.

Given the high prevalence of keratoconus in Saudi Arabia, improving public health awareness should be a priority. Dedicated awareness programs should be implemented at multiple levels. For example, collaborating with the Ministry of Education to introduce annual school screening programs is essential for early detection and prevention of complications. Additionally, community campaigns and health awareness initiatives will play a pivotal role in raising awareness and promoting better eye health practices. Social media platforms can also be utilized to reach a larger segment of the population effectively.

Furthermore, eye care professionals, including optometrists and ophthalmologists, should incorporate routine patient education focused on addressing allergies and discouraging eye rubbing. Increasing public knowledge about keratoconus, its prevalence, and its risk factors will facilitate earlier diagnosis and less invasive treatments, reducing the need for advanced interventions such as corneal transplants.

## Data Availability

The original contributions presented in the study are included in the article/supplementary material. Further inquiries can be directed to the corresponding author.
